# Would Cortisol Measurements Be a Better Gauge of Hydrocortisone Replacement Therapy? Congenital Adrenal Hyperplasia as an Exemplar

**DOI:** 10.1155/2020/2470956

**Published:** 2020-10-13

**Authors:** Peter C Hindmarsh, John W Honour

**Affiliations:** ^1^Departments of Paediatrics, University College London Hospitals, London, UK; ^2^Institute for Women's Health, University College London, London, UK

## Abstract

There is an increase in mortality and morbidity as well as poor quality of life in patients with congenital adrenal hyperplasia (CAH) and other causes of adrenal insufficiency. Glucocorticoid replacement therapy should aim to replace the missing cortisol as close as possible to the normal circadian rhythm using hydrocortisone. Dosing should be based on the individual's absorption and clearance of the drug. Adequacy of dosing should be checked using 24-hour profiles of plasma cortisol with samples drawn preferably every hour or at least every 2 hours. Measurement of cortisol should be the preferred method of assessing replacement therapy as it is over- and undertreatment with hydrocortisone, both of which can occur over a 24-hour period, which leads to the problems observed in patients with CAH and adrenal insufficiency.

## 1. Introduction

It is important to understand the normal pattern of cortisol production to manage the treatment of adrenal insufficiency. The term circadian rhythm is used in normal subjects to describe the differences in plasma cortisol concentrations recorded over 24 hours with high concentrations averaging 400–500 nmol/l from 06 : 00 to 08 : 00 h and low concentrations averaging 50 nmol/l measured around midnight. Plasma cortisol concentrations below 50 nmol/l are rarely seen in normal profiles over the 24-hour period. This is important because cortisol has permissive actions at low concentrations maintaining a number of important body functions: water clearance in the kidney, glucose homeostasis, bone metabolism, and catecholamine synthesis [1].

**Figure 1:**
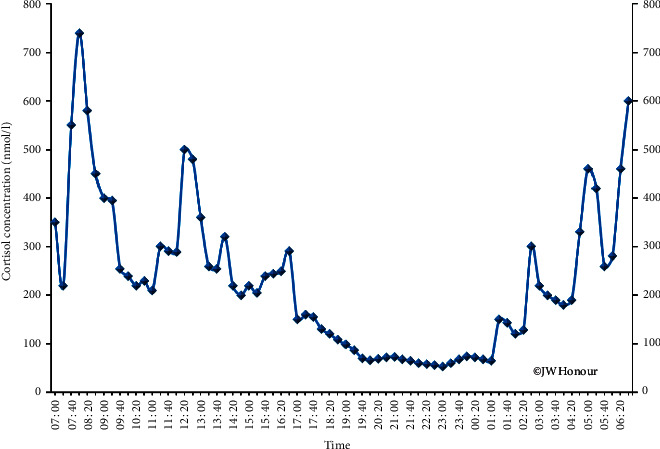
Circadian rhythm of plasma cortisol concentrations in a single normal male volunteer aged 55 years. Samples are obtained at 20-minute intervals (see [2]).

If more frequent sampling is undertaken every 20 minutes (Figure 1), a pulsatile pattern can be identified [3] with pulse frequencies between 70 and 90 minutes—an ultradian rhythm. The significance of such a rhythm is unclear given the average half-life of cortisol of 80 minutes [4]. Understanding cortisol rhythms and, in particular, the circadian rhythm is crucial to the interpretation of blood test results and to the treatment of disorders affecting cortisol synthesis.

The circadian rhythm probably plays an important role in synchronising the central and peripheral clocks that are so important in metabolism. The plasma cortisol concentrations are not the only part of this system that change during the 24-hour period, and we need to be mindful that differences in sensitivity probably operate between tissues as well as during the 24-hour period. Exogenous glucocorticoids, for example, are known to have a greater suppressing effect on endogenous production when given at night compared to that during the day.

Replacement therapies attempt to mimic the normal circadian rhythm [5, 6] but unlike, for example, thyroxine replacement, a single measurement does not provide information on the adequacy of replacement. Achieving a normal plasma cortisol concentration profile is a challenge with the available treatment modalities. The situation is complicated by the side effects of each adrenal or pituitary disorder where further hormone defects or excesses come into play notably with sex steroids and mineralocorticoids. We use cortisol replacement therapy in congenital adrenal hyperplasia (CAH) as a model to argue the case for more detailed assessments to be made of what is achieved with glucocorticoid replacement therapies and propose for discussion and further assessment that cortisol should become the prime measure to determine the adequacy of replacement in CAH and its role explored in other forms of adrenal insufficiency. At this stage, we can only relate to cortisol measurements as commercial assays for other glucocorticoids such as prednisolone and dexamethasone are unavailable.

Recent reviews of causes of mortality in CAH in children and adrenal insufficiency in adults [7–9] point to cortisol deficiency as the primary cause in many instances. Excluding emergency care protocols and the application of sick day rules, the risk of mortality probably reflects how well cortisol is replaced on a day-to-day basis. Standardised Mortality Rates are some eightfold higher in patients with pituitary ACTH deficiency on cortisol replacement compared to hypopituitary patients with no ACTH problem [10] who have Standardised Mortality Rates similar to the general population.

In addition, if we consider the list of problems that can be encountered by patients with CAH or any form of adrenal insufficiency, the vast majority of side effects relate to over- or undertreatment with glucocorticoids. Undertreatment during the 24-hour period can lead to unwanted effects such as hyperandrogenism. There are also data in children which point to the evolution of features of the Metabolic Syndrome, obesity, hyperinsulinism, hypertension, and hyperlipidaemia [11]. For adults with CAH, survey data confirm that the major long-term problem is weight gain [12] whilst osteoporosis is appearing in addition. Studies in older men without adrenal insufficiency suggest that higher trough but not peak cortisol concentrations are associated with an increase in the rate of loss of bone mineral density, highlighting the importance of different components of the cortisol profile in determining metabolic effects [1]. These observations would argue that 17 OHP and/or A4 cannot be used alone as markers in CAH for hydrocortisone replacement. Testosterone is also problematic because of the testicular source of most of this androgen. Those measures need to be interpreted with respect to how it is affected by cortisol, i.e., how much hydrocortisone has been given, when it was given, how long the cortisol lasts in the blood, and what concentrations of cortisol were attained.

## 2. General Points on Endocrine Replacement

**Figure 2:**
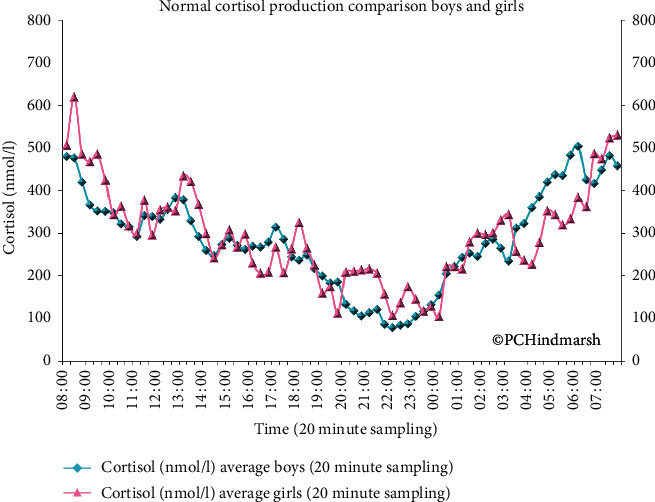
24-hour plasma cortisol concentrations in 28 short prepubertal boys (blue line) and girls (pink line) with normal endocrinology. Samples are drawn at 20-minute intervals. Data are shown as mean value at each time point (see [2]).

One of the main rules of endocrinology is to replace the hormones which are missing as close as possible to the way the body would naturally produce them. Hydrocortisone is the preferred glucocorticoid replacement therapy in CAH, but when using hydrocortisone replacement therapy, we need to consider the 24-hour pattern of cortisol production.  Figure 2 shows the mean plasma cortisol concentrations in a controlled study of mealtimes and sleep from 28 short normal prepubertal children aged between 5.5 and 11.9 years with samples drawn every 20 minutes for the 24-hour period. The concentrations can vary slightly between individuals but the pattern which is the circadian rhythm remains remarkably reproducible between individuals. There are several important points to note from this profile:

  The body is not without cortisol at any point in time although the values are the lowest at about 22 : 00 h (later in adults between 24 : 00 h and 02 : 00 h)
  From the low concentrations in the evening, the amount in the blood starts to rise progressively in peaks with a final peak in the morning between 06 : 00–08 : 00 h
  The increased cortisol concentration remains during the morning hours and only starts to decline from late afternoon to lower values in the late evening

If we mimic the physiological situation, then we need to take different doses of hydrocortisone throughout the day and night. How often hydrocortisone should be taken is determined by how long it takes to absorb the oral hydrocortisone and how fast cortisol is removed from the bloodstream. The peak plasma cortisol concentration following oral hydrocortisone occurs on average 60–90 minutes after ingestion and varies between individuals. Equally, although the half-life of hydrocortisone has a mean value of 80 minutes, an individual half-life can range from 40 to 225 minutes [13]. This means that someone with a half-life of 40 minutes would not do well with a three or four times per day regimen as each dose would only last 4 hours at most whereas the person with a half-life of 220 minutes would be overtreated on a three or four times per day regimen.

The circadian pattern is similar in adults and children. The peak cortisol is achieved around the same time 06 : 00–08 : 00 h, and the concentration attained is also very similar. A difference is when the lowest plasma cortisol concentration is achieved. For children, this occurs at 22 : 00 h whereas for adults it occurs a bit later at midnight in older adults and 02 : 00 h in younger adults [2, 14]. There are no gender differences.

From these data, the total production rate of cortisol by the adrenal gland is calculated to 8 to 10 mg/m^2^/day, which for an oral drug would translate into 10 to 12 mg/m^2^/day due to first pass metabolism in the liver as well as the enterohepatic circulation [2]. These figures are much less than doses used in the past based on radioactive isotope dilution studies [15, 16]. The combination of the total daily dose and the estimated percentage distribution of cortisol mentioned above allows for the best estimate of dose for the individual. It is likely that this will change depending on how the individual handles hydrocortisone which will be considered further below.

**Figure 3:**
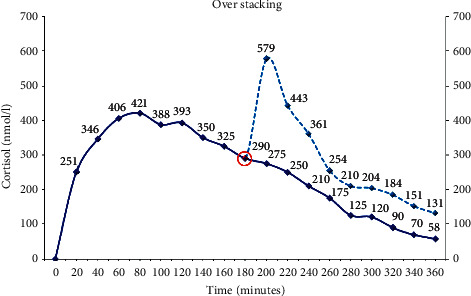
Hydrocortisone stacking in a prepubertal male aged 10 years with salt-wasting congenital adrenal hyperplasia on hydrocortisone dosing of 12 mg/m2/day and a 9-alpha fludrocortisone dose of 100 micrograms per day. A dose of hydrocortisone (7.5 mg) is given at time zero (time course depicted by the solid blue line). On a second occasion, a further lower dose (5.0 mg) (time course depicted by the dashed blue line) is given 180 minutes; later, the second dose stacks on top of the previous dose (see the circled point) giving a higher cortisol concentration than expected. Samples are drawn at 20-minute intervals Reproduced from Congenital Adrenal Hyperplasia: A Comprehensive Guide, Hindmarsh P and Geertsma K Elsevier, New York (2017).

The reason that timing and distribution are important relates first to our aim of getting the cortisol delivered to best approximate the normal circadian rhythm. The second reason is that if due consideration is not given to the pharmacology, it is possible to underreplace and equally to overreplace a phenomenon called “Stacking” (Figure 3). If a reasonable period of time is not allowed between doses, the cortisol concentration from the second dose becomes superimposed on that from the first and quite high concentrations can be attained without the individual realising.

### 2.1. Hydrocortisone Treatment

**Figure 4:**
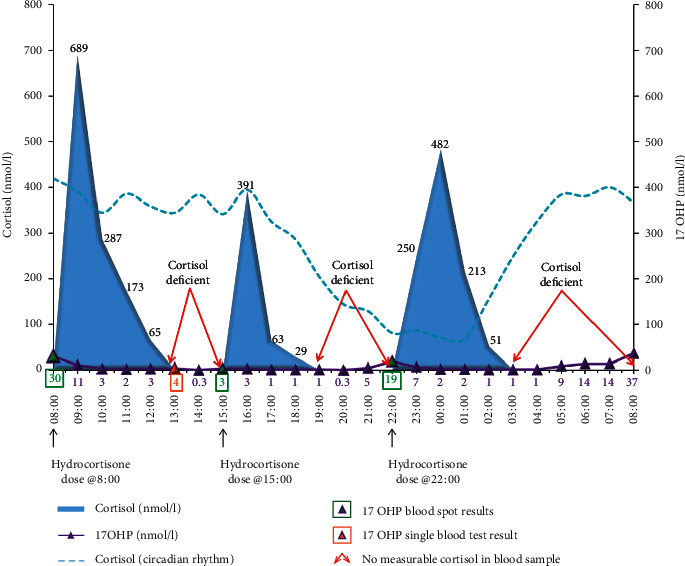
Plasma cortisol (filled area) and 17 hydroxyprogesterone (purple line with triangles) concentrations obtained after three oral doses of hydrocortisone given at 08 : 00 (7.5 mg), 15 : 00 (5.0 mg), and 22 : 00 h (5.0 mg) in a single 12-year-old pubertal female with salt-wasting congenital adrenal hyperplasia on hydrocortisone dosing of 12 mg/m2/day and 9-alpha fludrocortisone dose of 100 micrograms per day. Average circadian plasma cortisol concentrations from the 28 short normal children are shown as the blue dashed line. Note the periods of time without cortisol and the rise in 17 hydroxyprogesterone concentrations from 04 : 00 h. Blood spot 17 hydroxyprogesterone concentrations are shown in boxes (single sample in orange and several samples in green) for comparison. Blood sampling was at 1-hour intervals. Reproduced from Congenital Adrenal Hyperplasia: A Comprehensive Guide, Hindmarsh P and Geertsma K Elsevier, New York (2017).

Figure 4 shows the plasma cortisol concentrations attained after three oral doses of hydrocortisone at 08 : 00, 15 : 00, and 22 : 00 h in a patient with CAH. The highest plasma concentrations of cortisol at 689, 391, and 482 nmol/l are seen at 10 : 00, 16 : 30, and 24 : 00 h. The pattern will vary between patients in terms of peak height and peak width depending on the way they handle hydrocortisone [13]. The duration that cortisol or any drug or hormone remains in the circulation is affected by absorption, protein binding, metabolism (to inactive metabolites), and clearance by excretion of metabolites in urine [17]. Another important observation in Figure 4 is the periods where cortisol is below the limit of detection in the assay. In these periods, it is likely that the patient will suffer from the absence of the permissive effects of cortisol. Furthermore, there are periods where concentrations of cortisol are high which will impact on obesity, insulin resistance, and mental disturbances.

How frequent a drug should be administered is determined by the half-life of the drug in the circulation. For hydrocortisone, this is on average 80 minutes, so dosing would be required roughly once every 6 hours (three or four doses per day) with doses varied to mimic the circadian rhythm. Dose distribution needs to provide about 30% of the total daily dose between midnight and 06 : 00 h, 35% between 06 : 00 and 12 : 00 h, 20% between 12 : 00 and 16 : 00 h, and 15% at 16 : 00 h where there is a natural burst [2]. This dosing schedule allows for the natural drop in cortisol in the late evening before the midnight dose. Therapies such as prednisolone and dexamethasone have been used, but in paediatric practice, they have not gained widespread favour due to associated weight gain, growth suppression, and osteoporosis [18].

### 2.2. Treatment of Congenital Adrenal Hyperplasia

For some forms of adrenal insufficiency (Addison's disease for example), cortisol would be the preferred choice of marker for assessing cortisol replacement therapy as no steroid intermediaries are measurable in such situations. Congenital adrenal hyperplasia due to a deficiency in the enzyme 21-hydroxylase is the commonest form of CAH. The conventional treatment of the condition uses hydrocortisone as a replacement glucocorticoid along with 9-alpha fludrocortisone as the mineralocorticoid replacement when necessary. Hydrocortisone dosing schedules need to be individualised [13].

In assessing cortisol replacement in CAH, plasma 17hydroxyprogesterone (17OHP) and/or androstenedione (A4) have been used as markers for assessing “control.” The argument for this has been that the aim of therapy is to reduce adrenal androgen production, although that aim does not necessarily mean that cortisol replacement is adequate. Cortisol replacement should not be excessive due to risks of weight gain and other side effects although this may relate to historic datasets rather than more contemporaneous data [19, 20]. It still occurs with dosing based on suppressing 17OHP, particularly when the cortisol delivered is not measured [18].

As many of the side effects that we wish to avoid in CAH relate primarily more to over- and/or undertreatment with hydrocortisone rather than adrenal androgen excess, it is worth considering a change in our thinking towards placing cortisol at the centre of replacement therapy and using cortisol as the primary measure to ensure optimal cortisol concentrations which will normalise ACTH and androgen levels in CAH. The hypothalamic-pituitary-cortisol axis operates as a negative feedback system. When this closed loop is opened due to cortisol deficiency, getting cortisol replacement correct is key because if it is correct, all surrogate markers will normalise because of the feedback loop.

### 2.3. Cortisol as the Primary Measure

Plasma 17OHP and/or A4 concentrations have been used in CAH traditionally as surrogate markers of pituitary activity. The argument has been that the aim of therapy is to reduce the ACTH drive to the adrenal glands thereby reducing cortisol precursors and adrenal androgen production avoiding the androgen effects on genital development, skeletal maturation, hair development, and gonadal function. As an additional point, it could be argued that it will indicate how much hydrocortisone to give, although the caveat is that this is only from the adrenal androgen point of view. Given that the hormone missing is cortisol and this is replaced with cortisol (hydrocortisone), it seems odd that 17OHP and A4 are the markers used to determine the adequacy of replacement rather than cortisol itself. Several observations question these assumptions.

### 2.4. Suppression of 17OHP and A4 Compared to Cortisol Production

**Figure 5:**
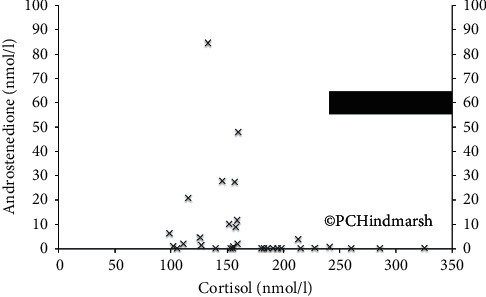
Relationship between plasma androstenedione concentration and 24-hour mean plasma cortisol concentration in 34 children with salt-wasting congenital adrenal hyperplasia showing step effect at 150 nmol/l. The black bar shows the normal range of 24-hour mean plasma cortisol concentrations (drawn at 20-minute intervals over 24 hours) in 80 normal adult individuals (aged between 60 and 70 years; see [2]). Reproduced from Congenital Adrenal Hyperplasia: A Comprehensive Guide, Hindmarsh P and Geertsma K Elsevier, New York (2017).

It has largely been assumed that suppression of 17OHP and A4 by cortisol acting on the pituitary occurs at cortisol concentrations that are similar to those encountered during the 24-hour period. This may not be the case [21]. First, the Inhibitory Concentration for plasma cortisol that produces a 50% reduction (IC_50_) in plasma 17OHP is 75 nmol/l which is well below the concentrations encountered in the normal circadian rhythm of cortisol. Second, there appears to be a threshold effect of cortisol on A4 (and similarly 17OHP) in prepubertal children with CAH (Figure 5). At a 24-hour mean plasma cortisol concentration of 150 nmol/l, plasma A4 becomes undetectable. However, this 24-hour mean plasma cortisol concentration is still lower than the range of 24-hour mean plasma cortisol concentrations encountered in profiles obtained from individuals without adrenal problems shown by the black bar in Figure 5. This means that suppression of the markers 17OHP and A4 can occur with circulating cortisol concentrations that are below the normal amount of cortisol encountered on a daily basis. Attaining the target of 17OHP and/or A4 suppression does not mean that ambient cortisol concentrations are adequately leaving the individual susceptible to symptoms and signs of cortisol deficiency even though the 17OHP/A4 would imply “adequacy of treatment.” This cannot always be seen in one-off blood tests due to the delay in the effect of cortisol on both these markers.

**Figure 6:**
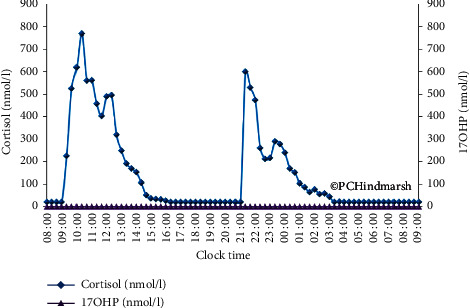
24-hour plasma cortisol (blue line and diamonds) and 17 hydroxyprogesterone (purple line and triangles) concentration profiles from a 16-year-old postpubertal girl with salt-wasting congenital adrenal hyperplasia receiving hydrocortisone twice daily (10 mg at 09.00 h and 7.5 mg at 21.00 h) for the previous 3 years. The dosing was hydrocortisone dosing 10 mg/m2/day and the 9-alpha fludrocortisone dose 100 micrograms per day. Blood samples are drawn at 20-minute intervals. Reproduced from Congenital Adrenal Hyperplasia: A Comprehensive Guide, Hindmarsh P and Geertsma K Elsevier, New York (2017).

Equally, a normal or suppressed 17OHP or A4 concentration does not tell us whether cortisol replacement is adequate. These concentrations could be achieved with just the right replacement or overtreatment. Neither 17OHP nor A4 allows for such fine discrimination.  Figure 6 illustrates the 17OHP obtained in an individual with CAH receiving hydrocortisone on a twice-daily basis. The high peaks of plasma cortisol occurring on a daily basis have led to the suppression of plasma 17OHP concentrations. This would not be identified with 17OHP measurements alone nor would the periods of time when there is no cortisol in the circulation (04 : 00–09 : 00 h and 15 : 00–21 : 00 h, nearly 50% of the day). This generates potentially, in the same individual, times of the day when there may be symptoms of over- or undertreatment. This adds to the uncertainty of using symptomatology to determine the adequacy of replacement alone as they can both be present in the same person. It just depends on what time of day is considered.

### 2.5. Factors Influencing 17OHP and A4

The situation is further complicated by the fact that 17OHP is influenced by several factors. 17OHP concentration varies in people without CAH during the 24-hour period, displaying a circadian rhythm like cortisol as does A4 [22, 23]. 17 hydroxyprogesterone varies during the menstrual cycle and is increased in women who have polycystic ovaries, a common finding in CAH. These observations point to the need to be careful of always attributing the adrenals as the source of the steroid.

17 hydroxyprogesterone and A4 will also be influenced indirectly by factors that alter cortisol metabolism such as puberty and alterations to the clearance mechanisms in the kidney and liver [4]. In addition, factors that influence cortisol binding globulin will impact indirectly by altering free cortisol in circulation. Oestrogen exposure from the oral contraceptive pill will increase [24] and hyperinsulinism will reduce plasma cortisol binding globulin concentrations. Even stressful procedures such as venepuncture or finger-prick testing will transiently raise plasma 17OHP concentrations.

### 2.6. Interaction of Cortisol and 17OHP and Mismatching of Values

**Figure 7:**
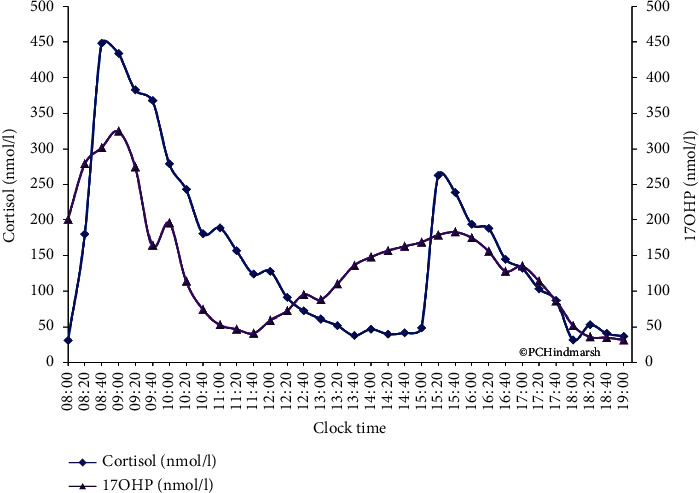
Temporal relationship between cortisol (blue line and diamonds) and 17 hydroxyprogesterone (purple line and triangles) in a 12-year old pubertal female with salt-wasting congenital adrenal hyperplasia following the administration of hydrocortisone at 08.00 h (5 mg) and 14.30 h (2.5 mg) (daily dose 11 mg/m2/day) showing the lag between the peak cortisol concentrations at 08 : 40 h and 15 : 20 h and effect on plasma 17 hydroxyprogesterone concentrations. Blood samples are drawn at 20-minute intervals. Reproduced from Congenital Adrenal Hyperplasia: A Comprehensive Guide, Hindmarsh P and Geertsma K Elsevier, New York (2017).

The interpretation of plasma 17OHP concentrations has, therefore, to be undertaken with knowledge of the dosing schedule of hydrocortisone and some estimate of how long cortisol is present from the administration of hydrocortisone. Even then the 17OHP alone will not accurately indicate the amount of cortisol in the blood.  Figure 7 shows the temporal relationship between plasma cortisol concentrations resulting from hydrocortisone administration and the change in plasma 17OHP concentrations. There is a lag between a change in cortisol in the blood and a corresponding change in 17OHP of approximately 60 minutes which means that following a peak plasma cortisol attained some 60–90 minutes after an oral dose of hydrocortisone, plasma 17OHP concentrations will only be lowered some 3 hours or so after the dose was taken. The figure also suggests a threshold effect for cortisol and 17OHP in that plasma 17OHP concentrations only tend to rise when plasma cortisol concentrations fall below 100 nmol/l implying that therapy should always ensure that at all times of the day, there is some cortisol measurable in the circulation. Incidentally, this is similar to the estimate of IC_50_ for cortisol on 17OHP.

These observations confirm that 17OHP merely follows the change in circulating cortisol concentrations in a classic negative feedback manner. Excessively high doses or peaks of cortisol will lead to shrinkage of the adrenal and marked suppression of 17OHP which will not recover before the next high dose is administered. This is seen with twice-daily regimens (Figure 6) where 17OHP is suppressed throughout the 24-hour period, yet the individual swung from excessively high peaks to periods of no cortisol around with symptoms of both cortisol excess (weight gain) and deficiency [5]. The implication of this is that if the cortisol replacement is correct, then 17OHP and/or A4 will follow. This taken with the side effect discussion would argue for cortisol to be the primary marker.

**Figure 8:**
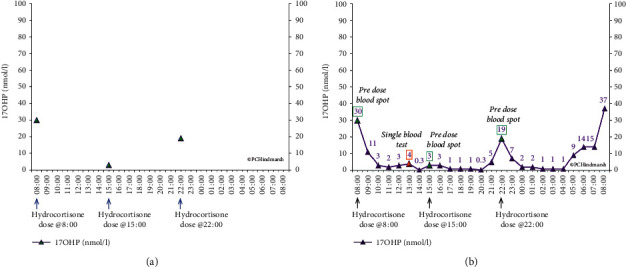
Full plasma 17 hydroxyprogesterone concentration profile (purple line and triangles) from the patient described in Figure 4 to illustrate the effect of sampling interval on the data obtained. Samples as (a) three time point spot samples (green triangles) before dosing with hydrocortisone and (b) how a single (orange triangle) or three (green triangles) time point samples do not reflect what is taking place over the 24-hour period. Blood samples are taken hourly to construct the profile. Reproduced from Congenital Adrenal Hyperplasia: A Comprehensive Guide, Hindmarsh P and Geertsma K Elsevier, New York (2017).

Without having a sound estimate of dose timing, it becomes difficult to determine what needs to be changed.  Figure 8(a) gives a picture in CAH of plasma 17OHP concentrations of measured prehydrocortisone dosing. The problem with this is that it is not possible to be sure what needs to be changed. For example, increasing the evening dose will not help the 22 : 00 h plasma 17OHP concentration as it is rising as the 15 : 00 h dose wears off. In addition, altering the 22 : 00 h dose is unlikely to make much difference to the early morning sample as the time course of hydrocortisone is such that the system will not be suppressed much after 03 : 00 h as cortisol will only be around in the circulation for 6 hours. Looking at the afternoon result for plasma 17OHP concentration in Figure 8(a) suggests that control may be satisfactory but it is not clear how this is achieved. Is the hydrocortisone dose at 08 : 00 h just right or perhaps too high? Is the timing of the doses correct as they could easily have run out of hydrocortisone earlier and about to escape from control?

More information can come from a full 24-hour profile of 17OHP (Figure 8(b)) which shows periods when the 17OHP looks good (only 6 of the 24 samples are above 10 nmol/l) but there are periods when the 17OHP looks less optimal. However, when we add the cortisol measurements, a different picture emerges (Figure 4). There are high peaks of cortisol which do not last to the next dose. The plasma 17OHP concentrations at 13 : 00 and 15 : 00 h are within the normal range, but at the same time, the cortisol is not measurable when, according to the circadian rhythm, there should be some around. So, although inferences on cortisol can be drawn, the answer to the question is that cortisol replacement optimal can only come from the measurement of cortisol.

## 3. Conclusions

In adrenal insufficiency, we are endeavouring to replace the missing hormone cortisol with the synthetic form, hydrocortisone. As the majority of the morbidity and to certain extent mortality in adrenal insufficiency relates to cortisol excess or insufficiency, it would make sense to focus on cortisol as the primary measure of the efficacy of replacement. The Endocrine Society Congenital Adrenal Hyperplasia Guidelines provide a useful overview of the condition [18]. The section on treatment and monitoring of therapy is short, probably reflecting the paucity of evidence from studies/trials in this area. In the absence of such information, we believe that we can still advance care using the principles of physiology and pharmacology outlined in this paper.

Cortisol replacement cannot be assessed in the same way as other hormones as there is no easy one-off measurement that will dictate over- or underreplacement, unlike thyroxine replacement, for example. Rather more detailed assessments using profile studies are going to be required periodically to more adequately define replacement therapy in whatever form, either tablets, pump therapy, or slow-release therapy.

## Data Availability

The data used in the study are available from the corresponding author.
